# Primary Human Blood Dendritic Cells for Cancer Immunotherapy—Tailoring the Immune Response by Dendritic Cell Maturation

**DOI:** 10.3390/biomedicines3040282

**Published:** 2015-12-02

**Authors:** Simone P. Sittig, I. Jolanda M. de Vries, Gerty Schreibelt

**Affiliations:** 1 Department of Tumor Immunology, Radboud University Medical Center, Radboud Institute for Molecular Life Sciences, GA Nijmegen 6525, The Netherlands; E-Mails: Simone.Sittig@radboudumc.nl (S.P.S.); Jolanda.deVries@radboudumc.nl (I.J.M.V.); 2 Department of Medical Oncology, Radboud University Medical Center, Radboud Institute for Molecular Life Sciences, GA Nijmegen 6525, The Netherlands

**Keywords:** human blood DC (dendritic cell) subsets, maturation, DC-based cancer vaccines, plasmacytoid DC, myeloid DC, CD141^+^ myeloid DC (mDC), CD1c^+^ mDC

## Abstract

Dendritic cell (DC)-based cancer vaccines hold the great promise of tipping the balance from tolerance of the tumor to rejection. In the last two decades, we have gained tremendous knowledge about DC-based cancer vaccines. The maturation of DCs has proven indispensable to induce immunogenic T cell responses. We review the insights gained from the development of maturation cocktails in monocyte derived DC-based trials. More recently, we have also gained insights into the functional specialization of primary human blood DC subsets. In peripheral human blood, we can distinguish at least three primary DC subsets, namely CD1c^+^ and CD141^+^ myeloid DCs and plasmacytoid DCs. We reflect the current knowledge on maturation and T helper polarization by these blood DC subsets in the context of DC-based cancer vaccines. The maturation stimulus in combination with the DC subset will determine the type of T cell response that is induced. First trials with these natural DCs underline their excellent *in vivo* functioning and mark them as promising tools for future vaccination strategies.

## 1. Introduction

Dendritic cells (DCs) are antigen-presenting cells that initiate and direct adaptive immune responses. Upon activation, they show a burst of antigen uptake, mature and travel to lymph nodes where they can drive naïve T cell activation and polarization. As such, they are considered excellent candidates for therapeutic cancer vaccines, which holds the great promise for re-calibration of the existing host-tumor interaction, tipping the balance from tumor tolerance towards tumor control. The goal of cancer immunotherapy is to elicit cytotoxic antigen-specific CD8^+^ T cell responses and thereby eliminate cancer cells via cellular immunity. Although numerous vaccination studies showed the immunogenicity of tumor-associated antigens introduced by dendritic cells [1,2,3], limited clinical success has been achieved with these vaccines. Likely, this is at least partially attributable to the local suppressive environment at the tumor site. Optimal DC maturation may help overcome the local suppression. Due to limited access to human tissue and blood DCs, much of our knowledge has come from mouse models and monocyte-derived DCs (moDCs). More recently, the understanding and the use of primary human DCs in cancer vaccination strategies has become within reach through efficient isolation techniques. In peripheral blood, at least two major types of DCs can be distinguished, namely myeloid DCs (mDCs) and plasmacytoid DCs (pDCs) [4]. Myeloid DCs can be subdivided by the surface expression of CD1c (BDCA-1) and CD141 (BDCA-3). Optimal stimulation of DCs used for cancer immunotherapy is essential to mediate immune responses that will be sufficiently robust and long-lasting to induce tumor regression and/or eradication and overcome the suppressive tumor microenvironment. Current efforts are aiming at finding the right stimulation of the DCs to induce such a robust and long-lasting response with the desired T helper type 1 (Th1) phenotype after administration to the patient. We review the current knowledge on the maturation of human DCs in the context of cancer immunotherapy. We reflect on the knowledge gained in studies with moDCs and then focus on human DCs from the blood, since in contrast to tissue DCs, these are accessible for DC-based vaccination strategies.

## 2. Dendritic Cell Maturation

The rationale behind DC-based cancer vaccines is the unique capacity of DCs to activate naïve and memory T cells and orchestrate an immune response. In the last two decades, we became to understand the life-cycle of a DC and how maturation primes them for induction of immune stimulatory responses. Immature DCs reside in peripheral tissues, where they can recognize and capture antigens. Upon activation, the DCs migrate to lymph nodes and other lymphoid tissues where they can initiate cellular responses. They present the captured antigens to CD4^+^ T cells via major histocompatibility complex (MHC) class II. Furthermore, DCs excel at cross-presentation, in which extracellular antigens are presented in MHC class I to CD8^+^ T cells and therefore allows cross-priming. The immune response initiated by the DCs depends on the signal that the DC receives upon encounter of the antigen. DCs can induce and regulate immunity against pathogens and tolerance against self-antigens and commensal microorganisms [5,6,7]. When no inflammatory or infection signal is sensed by the DC in the context of the antigen uptake, the DC will generally induce tolerance to what probably represents a self-antigen. On the other hand, DCs undergo a maturation process when—during antigen uptake—they are stimulated by pathogen-associated molecular patterns (PAMPs) via pattern-recognition receptors (PRRs) that are abundantly expressed on DCs [8]. Also proinflammatory cytokines such as IL-1, IL-6, IL-8 or TNF-α and damage-associated molecular pattern molecules (DAMPs) such as nuclear or cytosolic proteins can trigger specialized receptors or PRRs, respectively. PRRs on DCs include C-type lectin receptors, NOD-like receptors, RIG-I-like receptors and DNA receptors. These receptors detect pathogen specific proteins, lipids, carbohydrates and nucleic acids. A widely studied class of PRRs is the Toll-like receptor (TLR) family. To date, 10 different TLRs have been identified in humans that are either expressed on the surface (TLRs 1, 2, 4–6) or in endocytic compartments (TLRs 3, 7–10) and all recognize different molecular patterns (summarized in [9]). To induce maturation of DCs for cancer vaccines, agonists of TLRs are frequently used to imitate the encounter with a virus or bacterium. Dendritic cell maturation involves drastic phenotypical and functional changes which include an increase in the surface expression of MHC, migratory as well as co-stimulatory molecules, a burst in antigen-uptake and processing, and the induction of cytokine production. The term mature is often used not only functionally to designate immunogenic DCs but also to describe this phenotypic transformation. These changes in the DC prepare it for traveling to lymphoid organs where they prime antigen-specific T cells to become effector T cells.

The education of T cells by DCs is based on three fundamental signals. The first signal is the recognition of antigen by the T cell receptor (TCR) on T cells presented in the context of MHC molecules on DCs. This interaction mediates antigen specificity and T cell activation by downstream signaling of the TCR. The second signal is mediated by co-stimulatory and co-inhibitory molecules on DCs which further activate T cells and induce expansion or attenuate the T cell response, respectively [10]. The third signal determines the polarization of the T cells and is mediated by DC-derived cytokines. Dendritic cells are capable of educating naïve T cells into a range of effector cells including immunogenic CD4^+^ Th cells, cytotoxic CD8^+^ T cells as well as tolerogenic regulatory T cells (Tregs). In order for DC-based immunotherapy to elicit potent anti-tumor T cell responses, the administered DCs need to raise an immune stimulatory rather than tolerogenic T cell response. For example, the presence of interleukin (IL)-12 and type I interferons (IFNs) promotes the induction of Th1 cells, whereas IL-10 inhibits induction of Th1 cells and promotes the differentiation of Tregs [11,12]. In anti-viral responses, Th1 cells and antigen-specific cytotoxic CD8^+^ T cells are elicited to eradicate cells infected by the virus. This type of immune response is also highly desirable in anti-tumor strategies, in which the aim is to eradicate tumorous cells by cytotoxic CD8^+^ T cells.

## 3. Monocyte Derived DC Maturation for Cancer Immunotherapy

To date, mainly *ex vivo* generated moDCs have found application in the clinic. MoDCs are DCs differentiated *ex vivo* from monocytes. In about six days, the addition of the cytokines GM-CSF and IL-4 allows generation of a large number of moDCs [13,14] Several clinical studies comparing immature and mature moDCs proved that mature moDCs induced significantly better T cell and clinical responses than their immature counterparts. Jonuleit *et al.* [15] compared mature (maturation with PGE_2_, TNF-α, IL-1β and IL-6) and immature moDCs and found that only mature moDCs induced the expansion of syngeneic tumor peptide-specific CD8^+^ T cells that showed strong antigen-specific cytotoxicity. They also showed that while mature moDCs induced increased recall antigen-specific CD4^+^ T-cell responses in 87.5% of patients, immature moDCs did so in only 37.5% [16]. Superior immunological responses induced by matured moDCs were shown by a several studies performed by different groups and in different cancer types [17,18].

We know today that maturation is key to immunogenic DC activity and that steady-state DCs can induce tolerance [19,20] or T cell anergy or deletion [8,21]. Different ways to mature moDCs have been investigated with the goal to induce cellular immunity. Since IL-12 is a key driver of cellular immunity, different maturation cocktails were developed with a special attention to induce IL-12 secreting DCs. Factors used to mature moDCs include CD40 ligand (CD40L), tumor necrosis factor-α (TNF-α), IFN-α and IFN-γ. Direct activation by PAMPs can be mimicked using agonists for PRRs such as TLR3 ligand polyinosinic:polycytidylic acid (polyI:C), TLR4 ligand LPS, TLR7/8 ligand imiquimod (R848) and oligodeoxynucleotides (CpG) binding TLR9.

To better imitate an inflammatory environment, cocktails combining several factors have also been used. These factors include prostaglandin E2 (PGE_2_), IL-1β and IL-6. PGE_2_ induces maturation and strong CCR7 expression and migration capacity in moDCs and was widely used in initial maturation cocktails. However, encounter with CD40L-expressing cells following PGE_2_ stimulation limits the production of IL-12 and CCL19, a T cell attractant [22,23,24]. Furthermore, PGE_2_ induces the production of IL-12p40, but inhibits the active IL-12p70 heterodimer [25]. PGE_2_ also primes DCs for preferential interaction with Tregs; Tregs are attracted through elevated production of CCL22 even after the removal of PGE_2_ [26]. The addition of poyI:C and R848 to PGE_2_ resulted in potent IL-12 production and Th1 polarization while also maintaining CCL21-directed migration [27]. The advantage of combining PGE_2_ and TLR ligands has been supported by another study with the TLR7/8 ligand CL075 [28], but also partially challenged in a study where the presence of PGE_2_ during TLR ligation fully restored migratory capacity of moDCs, but left IL-12p70 production and activation of tumor antigen-specific cytotoxic T cells unaffected [29].

IFNs play a central role in the initiation of innate and adaptive immune responses and can be used alone or in combination with other factors to mature moDCs. Several studies show that IFN-α induces the differentiation and maturation of moDCs and also IFN-γ can be used to mature moDCs, leading to the secretion of large quantities of IL-12 and induction of Th1 cells [30,31,32,33,34].

## 4. Maturation of Plasmacytoid DCs in the Context of Cancer Immunotherapy

Plasmacytoid DCs are key effectors of innate immune responses due to their capacity to produce large amounts of type I IFNs IFN-α and IFN-β in response to bacterial or viral infections [35]. Plasmacytoid DCs mainly express TLR7 and TLR9 [36,37,38,39], recognizing ssRNA and CpG DNA, respectively. These intracellular TLRs therefore signal upon encounter with viral RNA, viral DNA or bacterial DNA. Both TLRs signal via MyD88 and induce maturation of pDCs. Plasmacytoid DCs can also be matured by ligation of CD40 by CD40L. T cell polarization induced by pDCs can vary and depends on cues such as differential TLR triggering [40]. The maturation and cytokine production of pDCs can be induced by TLR agonists such as R848 (TLR 7/8) and different classes of CpG (TLR 9) [35,41]. Upon activation with TLR agonists, pDCs upregulate MHC class I and II as well as co-stimulatory molecules and the lymph node homing receptor CCR7 [42,43] (summarized in [44]).

By now, a number of studies have shown that human pDCs can cross-present exogenous antigens to CD8^+^ T cells and that they even compare to mDCs in this capacity [43,45,46,47,48,49]. To which extend they are able to phagocytose cell-derived antigens and dead cells, is still to be established [50]. However, overall they appear to less efficiently take up antigens when compared to human mDCs [43,50]. Nonetheless, pDCs can take up soluble and particulate antigens [51] and receptors expressed by pDCs can be targeted (see targeting section). A recent study showed that pDCs can acquire membrane patches from cancer cell lines and present acquired antigens on MHC class I molecules; this mechanism may allow pDCs to present tumor-derived antigens, despite limited properties of phagocytosis [52].

**Figure 1:**
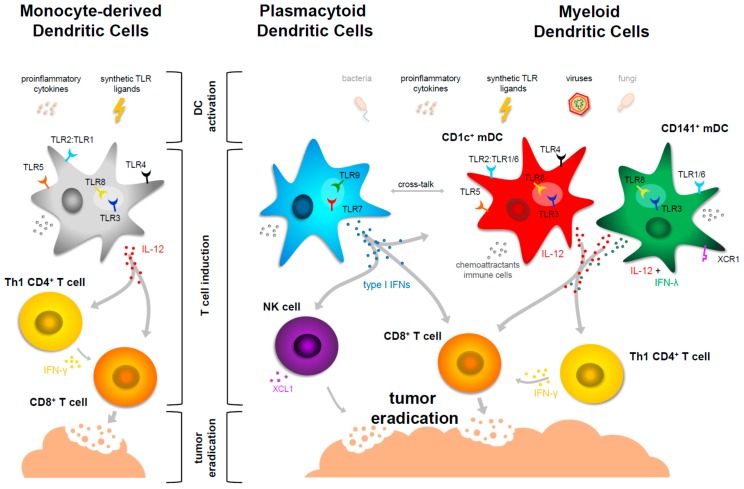
Maturation of moDCs and primary human DCs in the context of cancer immunotherapy. Stimulatory signals for human primary blood DCs include PAMPs from bacteria and viruses, but also proinflammatory cytokines. For cancer vaccine purposes, they can be matured by synthetic TLR ligands imitating natural ligands of TLRs. Monocyte-derived DCs used for DC-cell based cancer therapy are matured by proinflammatory cytokines or TLR ligands. TLR ligands mimicking viral infections—such as polyI:C (TLR3), R848 (TLR7/8) and CpG (TLR9)—have been proven most successful in inducing potent cellular responses. They trigger TLRs and induce maturation of the DCs; this increases expression of MHC and co-stimulatory molecules (not depicted) which support efficient T cell activation by the DCs. All subsets can cross-present antigen to CD8^+^ T cells and will direct T cell responses by soluble factors. Chemoattractants for immune cells are produced by all subsets. Plasmacytoid DCs are potent inducers of type I IFNs, which have pleiotropic effects on many cell types including T cells, NK cells and mDCs (cross-talk). Monocyte-derived DCs, CD1c^+^ mDCs and CD141^+^ mDCs produce IL-12, supporting CD4^+^ T cell skewing towards a T helper type 1 (Th1) phenotype. Mature CD141^+^ mDCs also produce IFN-λ, further supporting Th1 skewing. NK cells, activated produce chemokine (C motif) ligand 1 (XCL1), a chemokine sensed by CD141^+^ mDCs expressing its receptor chemokine (C motif) receptor 1 (XCR1). NK cells thereby attract CD141^+^ mDCs that are equipped to take up and (cross-) present dead cell material and initiate adaptive cellular T cell responses. Together, these functions of mature human moDCs and blood DCs allow the induction of potent cellular anti-tumor responses.

The large amounts of type I IFNs that are produced by pDC upon activation, have pleiotropic effects on the immune system and initiate and facilitate innate immune responses and an anti-viral state, but also support adaptive anti-tumor responses. pDCs are able to quickly respond to viral infection with an extensive ER compartment that facilitates high-capacity secretion of type I IFNs as well as pre-synthesized stores of MHC class I and II to rapidly activate CD4^+^ and CD8^+^ T cells [46]. The type I IFNs not only promote their own surivival as well as phenotypic and functional activation [53], but they also induce the upregulation of MHC class I on all cell types [54], activate macrophages and natural killer (NK) cells, and are critical for the activation and survival of both CD4^+^ and CD8^+^ T cells [55,56,57,58]. It has been shown in mice that type I IFNs boost cross-priming of antigen by DCs eliciting strong CD8^+^ T cell responses [59]. Importantly, the presence of IFN-α can restore the immunogenic capacity of human tolerogenic DC [60] and type I IFNs also enhance the maturation of mDCs [61] (discussed below). Therefore, the IFNs can help skew responses towards a Th1 phenotype and are relevant in the context of anti-tumor responses. Figure 1 summarizes pDC functions in the context of cellular immunity.

The fact that pDCs are able to induce strong anti-viral responses mediated by effective cytotoxic CD8^+^ T cell responses, already makes them promising candidates for inducing effective anti-tumor responses. Plasmacytoid DCs can infiltrate several human tumor types including ovarian cancer [62], breast cancer [63], head and neck cancer [64] and melanoma [65]. However, the immune inhibitory microenvironment of the tumor inhibits pDC differentiation and maturation [66,67]. Anti-tumor responses induced by pDCs have been reported in animal models injecting TLR matured pDCs into tumors lead to effective and systemic antitumor immunity in a mouse melanoma model [58]. In humans, both intra-tumoral injection of CpG (basal cell carcinoma and melanoma) [68] or topical application of R848 (basal cell carcinoma) [69] can overcome the inhibition exerted by the tumor microenvironment and induce type I IFN responses as well as tumor regression. In mice, depletion of pDCs shows a reduction in the frequencies and numbers of antigen-specific CD8^+^ T cells [42]. Although type I IFN production was not impaired and probably taken over by other cell types, involvement in T cell recruitment by pDCs via a soluble factor is suggested [42]. Therefore, pDCs exert relevant tasks for anti-tumor responses beyond the production of type I IFNs.

Importantly, steady-state pDCs induce tolerance rather than an immune stimulatory response. The induction of T cell anergy and IL-10 producing Tregs by immature pDCs has been described in different diseases and tumors (summarized in [42]). For example, pDCs exposed to the tumor microenvironment of malignant human ovarian epithelial tumor cells induced IL-10-producing Tregs rather than T cell activation, thus promoting tolerance instead of anti-tumor immunity [62]. In 2004, it was found that pDCs mediate tolerance to harmless inhaled antigen by inducing Tregs that suppress the generation of effector T cells by DCs [70]. To prevent tolerance, it is therefore important to induce maturation of pDCs in DC-based cancer vaccination strategies.

## 5. Maturation of Myeloid DCs in the Context of Cancer Immunotherapy

CD1c^+^ and CD141^+^ mDCs share some characteristics among which is the important capacity of both myeloid DC subsets to produce IL-12 upon TLR3 or TLR8 stimulation [71]. This enables the generation of Th1 CD4^+^ T cells and the priming of naïve CD8^+^ T cells.

Both mDC subsets are already equipped with high immune stimulatory capacities without any maturation and especially express high levels of MHC molecules, but also co-stimulatory receptors [72,73,74]. While MHC expression is barely affected, TLR ligation with polyI:C or R848 significantly increases the expression of co-stimulatory molecules such as CD40, CD80, CD83 and CD86 as well as the essential lymph node homing receptor CCR7 (summarized in [44]) [71]. CD1c^+^ mDCs express TLRs 1-6, TLR 8 and TLR10 [39,71]. TLR3 recognizes dsRNA, an intermediate of viral replication. TLR 8 (and 7) recognizes ssRNA. TLR1, TLR2 and TLR6 are triggered by bacterial lipoprotein and peptidoglycans, whereas TLRs 4 and 5 recognize the bacterial wall components lipid A of lipopolysaccharide and flagellin, respectively. TLR2 is also involved in the recognition of fungi. CD1c^+^ mDCs are therefore equipped to respond to bacterial and fungal PAMPs and to a lesser extend also to viral PAMPs. Upon TLR ligation, CD1c^+^ mDCs can produce a variety of cyto- and chemokines including IL-1α, IL-1β, IL-6, IL-8, IL-12, CCL3, CCL4, chemokine (C–C motif) ligand 5 (CCL5), chemokine (C motif) ligand 10 (CXCL10), TNF-α [71,72,75]. CD141^+^ mDCs possess a more limited repertoire of TLRs as well as cyto- and chemokines compared to CD1c^+^ mDCs. CD141^+^ mDCs express TLR1, TLR3, TLR6, TLR8 and TLR10 [71,73,76] and especially TLR3 is uniquely highly expressed as compared to CD1c^+^ mDCs and pDCs. TLR3 recognizes retroviral double-stranded RNA, which points to an anti-viral function of CD141^+^ mDCs. Upon TLR3 stimulation, CD141^+^ mDCs produce differing levels of IL-6, IL-8, IL-12, CCL5, CXCL10, TNF-α, IFN-β, and IFN-λ [49,71,73,77,78,79].

The chemokines expressed by both mDC subsets are able to attract and activate T cells. CCL5 is implicated in chemotaxis and activation of and array of immune cells including T cells and NK cells [58,80,81]. CXCL10 is a potent chemoattractant for activated T cells in mice [82]. IL-8 is a central chemoattractant stimulus for immune cells. Therefore, both mDC subsets play a role in recruiting and activating immune cells to the sight of inflammation.

In addition to TNF-α and IL-6, cytokines produced by both mDC subsets, CD1c^+^ mDCs produce two other mediators of inflammation, namely IL-1α and IL-1β. On the other hand, CD141^+^ mDCs exclusively produce IFN-λ [78], a cytokine associated with anti-viral effects. Furthermore, human CD141^+^ mDCs can produce IFN-β upon TLR3 stimulation [71] and in a humanized mouse model, CD141^+^ mDCs produced significant levels of IFN-α [83]. This capacity to produce type I IFNs, once more underline their anti-viral capacities.

Importantly, IL-12 can be produced by both mDC subsets. Initially, it has been assumed that CD1c^+^ mDC produce more IL-12 than CD141^+^ mDCs [71]. Some studies have shown that in order to induce strong IL-12 responses in human and mouse DCs, both an innate trigger such as TLR ligation and a second trigger like ligation of CD40 by CD40L on T cells is needed [71,78,84]. A synergistic effect in the induction IL-12 and of potent Th1 responses by different combinations of TLR ligands has been described for mouse DCs and human moDCs [27,85]; examples include the combination of R848 with LPS or polyI:C. More recently, it has been shown for CD1c^+^ mDCs, that the combination of the TLR ligands R848 and LPS can trigger significant IL-12 production [72]. In the case of CD141^+^ mDCs, polyI:C stimulation of whole blood or a cocktail of polyI:C together with for example IFN-γ, TNF-α, IFN-α, and IL-1β for isolated cells, was shown to induce significant levels of IL-12 in CD141^+^ mDCs [71,73,77]. In a recent study [86], we support the notion that a single stimulus is not sufficient to induce high IL-12 production, especially for CD141^+^ mDCs. Similar to the findings in moDCs [27,85], we found that the combination of polyI:C and R848 can trigger substantially higher IL-12 secretion by both human mDC subsets than each stimulus alone [86].

It is interesting to note that both CD1c^+^ and CD141^+^ mDCs react strongly and in a similar way to polyI:C [71,86], although the expression levels of TLR3 are much higher in CD141^+^ mDCs than in CD1c^+^ mDCs [71]. Likely, other receptors for polyI:C contribute to the response in one or both of the mDC subsets. The synthetic dsRNA analog is a ligand for multiple pathogen recognition receptors, and besides TLR3 also triggers cytosolic RIG-I-like receptors that are expressed by mDCs [87,88]. Perrot *et al.* [89] suggest in a study on mDCs and NK cells that both TLR3 stimulation as well as RIG-I-like receptor ligation is needed for IFN-γ induction by mDCs.

Both blood mDC subsets can cross-present soluble and tumor lysate-derived exogenous antigens to CD8^+^ T cells. In contrast to mouse DC subsets, in which CD8α^+^ DC seem to be clearly superior cross-presenters as compared to CD8α^−^ DCs, for human mDCs the situation is less clear cut. While some publications suggest that the different human blood DCs subsets compare in their cross-presentation capacity [43,74], several studies show superior cross-presentation capacity of CD141^+^ mDCs and put them forward as the human counterparts of mouse CD8α^+^ DCs [73,77,79,90,91]. The type and size of the antigen as well as the compartments the antigen is targeted to probably underlie these differing outcomes. For example, targeting antigens to early endosomal compartments in CD141^+^ mDCs, diminishes their superior cross-presentation capacities [74]. Importantly, CD141^+^ mDCs in these studies are activated with polyI:C, which boosts their cross-presentation capabilities. While tissue mDCs (tonsil, dermis) do not need maturation to cross-present [79,92,93], blood mDC cross-present only if matured with polyI:C or a combination of pI:C and R848 [43,71,73,77,79,93].

Just as for pDCs, the response mDCs will induce depends on the cues they receive from their environment. As an example, an immunoregulatory phenotype and function was demonstrated for CD1c^+^ mDCs after bacterial stimulation with *E. coli* [94]. However, multiple studies demonstrate that both matured blood CD1c^+^ and CD141^+^ mDCs are equipped to induce Th1 CD4^+^ T cell and CD8^+^ T cell responses [71,72,73,79,86,93]. Segura *et al.* [93] show, that in contrast to lymph node resident and migratory DCs, blood DCs induced efficient Th1 polarization, but poor Th2 polarization.

CD141^+^ mDCs are well equipped to take up material from dead and necrotic cells for subsequent cross-presentation of derived antigens to CD8^+^ T cells. Like mouse CD8α^+^ DCs, they almost exclusively express the CLR CLEC9A, which is an endocytic receptor involved in the sensing and presentation of antigens derived from necrotic cells [95,96]. PolyI:C activated human CD141^+^ mDCs were shown to cross-present antigen from necrotic cells [73,77]; although both mDC take up dead cell material, only CD141^+^ mDCs could cross-present antigen efficiently to CD8^+^ T cells [73]. 

Another link between human CD141^+^ mDCs and mouse CD8α^+^ DCs is the chemokine (C motif) receptor 1 (XCR1), which is exclusively expressed by these subsets [90,91]; it is the receptor for the chemokine XCL1 that is produced especially by activated NK and CD8^+^ T cells. Activated NK and CD8^+^ T cells therefore attract the subset specialized in cross-presenting dead cell material [90]. XCR1 knock-out mice have decreased early CD8^+^ T cell responses to the intracellular pathogenic bacteria *Listeria monocytogenes* infection [91].

In mice, CD8α^+^ DCs produce IL-12, excel at cross-presentation and polarize naive CD4^+^ T cells toward the Th1 cell phenotype, whereas CD8α^−^ DCs preferentially induce Th2 cell responses [97]. As discussed above, comparable characteristics of CD141^+^ mDCs with mouse CD8α^+^ DCs suggest them to be excellent targets for DC-based cancer immunotherapy [77,79,90,91]. However, the functional division between the two human mDC subsets is not as outspoken as in mice. Human CD1c^+^ and CD141^+^ mDCs can both produce large amounts of IL-12 and are both well capable to cross-prime CD8^+^ T cells [49,71,72]. Figure 1 summarizes the functions of primary human blood DCs in the context of cellular immunity. Whether it is feasible to isolate the rare subset of CD141^+^ mDCs from apheresis material for *ex vivo* maturation and antigen loading and subsequent re-injection into patients, remains to be shown. CD1c^+^ mDCs, on the other hand, are much more abundant and therefore easier accessible in sufficient numbers and have been used in first clinical studies (see section below).

## 6. Human Primary DC Subsets as Cancer Vaccines

Primary DCs are hypothesized to be stronger inducers of anti-cancer responses than moDCs in cell-based vaccination strategies since they differentiate *in vivo* and require only short *ex vivo* handling, a process that might negatively affect DCs. Especially IL-4, required for the transition of monocytes into immature DCs may hamper their capability to migrate [98,99]. The first clinical studies utilizing primary blood DCs have recently been conducted, demonstrating the safety and efficacy of primary blood DCs in cancer immunotherapy. Late stage melanoma patients vaccinated with *ex vivo* activated and antigen-loaded pDCs showed remarkably improved overall survival compared to matched control patients treated with standard chemotherapy as first-line treatment [100]. Due to limited availability of clinical grade TLR ligands that activate pDCs, the pDCs in this trial were matured with FSME, a preventive vaccine against the tick-borne encephalitis virus. PDCs matured with FSME demonstrate a mature phenotype and produce large amounts of type I interferons [101,102].

By now, two trials have been conducted with CD1c^+^ mDCs. Prue *et al.* [103] vaccinated prostate cancer patients with immature, tumor-antigen loaded CD1c^+^ mDCs. Although the administration was safe, no tumor specific immunological responses were reported. In another recent trial using CD1c^+^ mDCs, three out of 14 vaccinated metastatic melanoma patients showed functional tumor-specific T cells in peripheral blood and post-treatment skin tests, coinciding with improved progression-free survival and objective clinical responses [104]. In this trial, the CD1c^+^ mDCs were cultured with GM-CSF; a stronger maturation stimulus such as a TLR3 ligand polyI:C or TLR7/8 ligand R848 would likely even increase the number and magnitude of responses. Since clinical-grade TLR ligands are difficult to obtain [101,105], we recently characterized RNA stabilized in a formulation with protamine as a possible clinical grade TLR ligand, which induced a mature phenotype and secretion of type I IFNs and IL-12 by pDCs and CD1c^+^ mDCs, respectively [106]. Clinical studies utilizing protamine-mRNA matured pDCs and CD1c^+^ mDCs are currently ongoing in patients with prostate cancer and melanoma.

For proper CD8^+^ T cell activation and memory formation, type I IFNs, IL-12 and IFN-γ are important players [107]. Matured CD1c^+^ and CD141^+^ mDCs can produce IL-12 and matured pDCs produce the highest amounts of type I IFNs, and all three subsets can induce IFN-γ production by Th1 and other cells. Therefore, simultaneous activation of several DC subsets would probably more efficiently raise a strong cytotoxic anti-tumor response. Interestingly, pDCs and mDCs can interact with each other and synergize via bi-directional cross-talk and this cross-talk is instrumental in increasing anti-tumor immune responses (summarized in [108]). Examples include the upregulation of co-stimulatory molecules on mDCs after selective pDC activation [61] or the recruitment and important role of mDCs in pDC mediated anti-tumor responses [58]. Therefore, co-administration of properly matured pDCs and CD1c^+^ mDCs may induce even more potent anti-tumor responses than pDCs or mDCs alone. As protamine-RNA complexes efficiently mature both pDCs and CD1c^+^ mDCs, they harbor the potential for the combined maturation and use of these subsets, thereby exploiting their functions simultaneously. Considering the excellent capacities of mature CD141^+^ mDCs to prime CD8^+^ T cell responses and their similarity to mouse CD8α^+^ DCs, utilizing also this subset strongly recommends itself.

## 7. Inducing Maturation of DC Subsets by *in Vivo* Targeting

To avoid *ex vivo* handling and its possible negative effects on the cells and also to move towards an off-the-shelf approach for activating anti-tumor immunotherapy, several research groups pursued the idea to target DCs *in vivo*. Besides the delivery of antigen, the co-administration of maturing agents is pivotal in the context of therapeutic DC-based vaccinations, as mouse models have shown that the lack of a maturation factor or an adjuvant can induce tolerance or a humoral response rather than the desired CD8^+^ T cells response [21,109]. Systemic administration of adjuvants in mice have shown anti-tumor effects and acts at least partially via DC maturation [110]. However, this systemic administration can cause severe side-effects, and co-localization of target antigen and adjuvant drastically improve anti-tumor responses [111]. Approaches for targeted co-delivery of antigen and maturation agent include coupling antigen to adjuvant and possibly to an antibody or co-encapsulation in degradable particles. Particles are very versatile and can carry antibodies on the surface to target specific cell subsets and several components that become released continuously after intake and digestion by the target cell.

Initial efforts strived to target DEC-205 [8] and by now, the efficacy of targeting DCs via DEC-205 is shown in numerous mouse and human *ex vivo* models (summarized in [112]). Since then, many other options have been explored for DC targeting. Particles co-delivering antigen and adjuvant have been successfully targeted to mouse and human DCs; the co-delivery of TLR ligands herein strongly enhanced adjuvanticity [113]. Cargo is often targeted to uptake receptors, deploying their capacity to deliver their ligands into the cell. Cohn *et al.* [114] give an overview of endocytic receptors that have been studied in primary human DC subsets, indicating the intracellular compartment they target and their ability to deliver antigen to MHC class I and/or MHC class II pathways. These receptors with varying cell type specificity are CD11c, CD32 (FcγIIa), CD40, CD205 (DEC-205), CD206, CD207 (Langerin), CD209 (DC-SIGN), CD303 (BDCA-2) [115], CLEC4A (DCIR), CLEC9A (DNGR1) and Dectin-1.

Depending on the receptor, a certain subset or group of cells will be targeted. A combination of several targets would simultaneously make use of specific characteristic of the targeted subsets. Also depending on the targeted receptor, the cargo such as antigen and a possible maturation factor, will be enriched in certain compartments of the cell. This can affect the availability of the antigen to MHC class I presentation pathways and the maturation agent to its cognate receptor. Some receptors route their cargo to early endosomes in which antigen is slowly digested, leading to prolonged MHC class I presentation to CD8^+^ T cells [74,116,117]. In CD141^+^ mDCs, targeting late endosomal compartments via DEC-205 however favors cross-presentation, while targeting early endosomal compartments via CD11c or CD40 diminishes their superior cross-presentation capacities compared to other human DC subsets [74]. This hints of a more efficient escape of antigens from late endosomes into the cytosol in CD141^+^ mDCs.

Nanoparticles containing antigen and adjuvant and covered with antibodies that bind different uptake receptors (DEC-205, DCIR, CD303 and Dectin-1) or the Fcγ receptor CD32, can all be delivered to pDCs inducing maturation, cytokine production and delivering the antigen to MHC I and II pathways [115,118] Fcγ receptors can also be targeted on CD1c^+^ as well as CD141^+^ mDC. While antigen is more efficiently taken up by CD1c^+^ mDCs, only CD141^+^ mDCs show improved cross-presentation by Fcγ receptor targeting [119]. Targeting antigen to mouse CD205 and simultaneously maturing DCs protected against a subsequent challenge with tumor cells [120]. Furthermore, vaccination with the same strategy after establishment of a tumor could inhibit further outgrowth of the tumor. Two mouse model studies underline the importance of CD8α^+^ DCs—and, therefore, CD141^+^ mDCs—as powerful targets for DC-based vaccination strategies. Targeting CD8α^+^ DCs via XCR1 (fusion protein of influenza A antigen and the XCR1 ligand XCL1), induced CD8^+^ T cell responses, Th1 CD4^+^ T cell responses and importantly protected mice against a lethal challenge with influenza A virus [121]. XCR1 is exclusively expressed on mouse CD8α^+^ DCs and human CD141^+^ mDCs. Importantly, targeting CLEC9A on CD8α^+^ DCs efficiently induced CD8^+^ T cell responses [122,123] and when combined with an adjuvant could both prevent the development as well as mediate the eradication of B16 melanoma lesions. Antigen targeted to CLEC9A on human CD141^+^ mDCs is presented to both CD4^+^ and CD8^+^ T cells and induces a strong IFN-γ response when combined with a maturation agent [124]. The plasticity of mDC and the importance for the use of adjuvants was nicely demonstrated in a mouse study in which CLEC9A was targeted either with antigen alone or combined with different adjuvants. Antigen delivery alone induced Tregs, whereas curdlan induced Th17 cells and polyI:C strong IL-12 dependent Th1 response [125].

## 8. Conclusions and Future Perspectives

In recent years, more insight has been gained about the best possible maturation of the subsets and their differences in terms of antigen-processing and -presentation, cytokine production and T cell stimulatory capacity. We know that the use of proper adjuvants is crucial in inducing potent T cell responses. Mouse models are a necessary and valuable tool to gain many insights into the functioning of vaccine mechanisms. A major challenge, however, remains to translate DC-based vaccine strategies developed in mice to humans. There are substantial differences between the mouse and human immune systems and the exact equivalent of each mouse DC subsets in humans is not yet established. For example, the use of mouse models to study and select adjuvants for human vaccines and targets for targeting strategies is limited because the pattern of expression of TLRs and various receptors per DC subset can significantly differ between the two species. Overall, the division of labor between human blood CD141^+^ DCs and CD1c^+^ mDCs appears less sharply demarcated than between CD8α^+^ and CD8α^−^ DCs in the mouse. CD1c^+^ mDCs should not be easily discarded for DC-based vaccination strategies, since—unlike CD8α^−^ DCs—they can produce large amounts of IL-12 and are well capable to cross-prime CD8^+^ T cells [49,71,72]. Therefore, further studies into the functional specializations of different DC subsets are called for and ongoing.

Analysis of the immune response to successful human viral vaccines that induce potent CD8^+^ T cell responses could help further determine the mechanisms that control immune responses to vaccination and identify predictors of vaccine efficacy. A successful example of such an approach is a study on the yellow fever vaccine. The researchers found that the potent CD8^+^ T cell responses and protection induced by this vaccine are at least in part attributed to autophagy induced by the vaccine that boosts cross-presentation of DCs [126].

Another crucial question at present is whether targeting specific DC subsets is more efficient than using well-formulated protein-based or particle-based vaccines with potent TLR adjuvants. Future studies need to compare these approaches using antigens from tumors that are more physiological antigens than ovalbumin, as these provide greater predictive value before advancing to human studies.

Since a kaleidoscope of factors determine the actual final outcome of a response, including *in vivo* factors that are not present *ex vivo*, we will only know which choice of antigens, combination of adjuvants, combination of DC subsets and concomitant treatments such as chemotherapy, ablation therapy or agents releasing local immunosuppression at the tumor site is best after testing them *in vivo*. Antigens should be immunogenic and ideally only be highly expressed by the tumor. Neoantigens are antigens only expressed by tumor cells and are created by DNA mutations in tumor cells. Such antigens can induce strong immune responses, since they are recognized as foreign and as such T cell specific for these antigens will not be affected by tolerogenic mechanisms that inhibit specific T cells that recognize self-antigens. Importantly, the combination of DC-based cancer vaccines with agents inducing temporary tumor regression or releasing local immunosuppression at the tumor site holds the promise of making the successful induction of T cell responses strong enough to tip the balance towards cancer immunity [127,128]. As targeted therapies can induce rapid tumor regressions and a decrease in tumor-associated immunosuppression, they may afford a favorable window for immunotherapy to achieve more potent cellular immunity [129]. Checkpoint inhibitors such as ipilimumab (anti-CTLA-4 antibody), nivolumab (anti-programmed cell death 1 (PD-1)) or pembroluzimab (anti-PD-1 receptor) have already successfully entered the clinic as anti-cancer treatments. A small first study combining DC vaccination with ipilimumab treatment for melanoma suggests that such combinations can be beneficial in treating cancer patients [130]; the results of further combination studies with DC vaccines are eagerly awaited.
